# Cost effectiveness analysis comparing varying booster intervals of vaccination policies to address COVID-19 situation in Thailand, 2023

**DOI:** 10.1371/journal.pone.0310427

**Published:** 2024-09-17

**Authors:** Chayanit Mahasing, Rapeepong Suphanchaimat, Pard Teekasap, Natthaprang Nittayasoot, Suphanat Wongsanuphat, Panithee Thammawijaya

**Affiliations:** 1 Division of Epidemiology, Department of Disease Control, Ministry of Public Health, Nonthaburi, Thailand; 2 International Health Policy Program, Ministry of Public Health, Nonthaburi, Thailand; 3 Faculty of Business Administration and Technology, Stamford International University, Prawet, Bangkok, Thailand; Istanbul University-Cerrahpasa, Cerrahpasa Medical Faculty, TÜRKIYE

## Abstract

The COVID-19 booster immunization policy is cost-effective, but evidence on additional booster doses and appropriate strategies is scarce. This research compared the cost-effectiveness of annual, twice-a-year, and biennial booster dose policies. We performed stochastic modeling using compartmental susceptible-exposed-infectious-recovered models and a system dynamic model. We evaluated four policy scenarios: (1) hypothetical no-booster immunization policy; (2) twice-a-year vaccination policy; (3) annual vaccination policy; and (4) biennial vaccination policy. In addition, we conducted a one-way sensitivity analysis by adjusting R0 from 1.8 to 3.0 in all scenarios (epidemic stage) and by decreasing the vaccination cost by 50% at the end of the first year to reflect the current policy direction to enhance domestic vaccine production. Compared to non-booster policies, all three booster strategies reduced the number of cases, hospital admissions, and severe infections remarkably. Without a booster, total cases would reach 16,220,615 (95% confidence interval [CI] 6,726,550–29,661,112) by day 1,460, whereas, with a twice-a-year booster, the total cases would reach 597,901 (95% CI 526,230–694,458) in the same period. Even though the no booster scenario exhibited the lowest cost by approximately the first 500 days, by day 1,460 the biennial booster scenario demonstrated the lowest cost at 72.0 billion baht (95% CI 68.6–79.4 billion). The most cost-saving policy was the biennial booster scenario. The annual booster scenario also stood as a cost-effective option for most outcomes. In the epidemic stage and in an assumption where the vaccination costs dropped, all booster policies became more cost-effective or cost-saving compared with the main assumption. This study underscores the significance of the COVID-19 vaccine booster policy. Implementing policies should take into consideration cost-effectiveness, feasibility, and public communication.

## Introduction

At the end of 2022, approximately 665 million COVID-19 cases had been documented worldwide [1] and over 6.6 million deaths had been reported globally [2]. The development of vaccines became an essential weapon for combating the COVID-19 pandemic. It is universally accepted that COVID-19 vaccinations offer huge benefit in decreasing infection incidence, severity, hospitalization, and fatality rates across various groups in the population [3–6]. However, the combination of reduced vaccine efficacy against infection in the context of Omicron sub-lineages and the gradual waning of the effectiveness after one or two shots after vaccine administration has emphasized the need for a booster immunization policy [7].

A series of clinical trials provided huge amount of information on COVID-19 vaccine effectiveness. Two doses of the main mRNA and adenovirus vaccines offered over 75% protection against severe disease, hospitalization, and mortality for six months against the Alpha and the Delta SARS-CoV-2 variants. With the introduction of the Omicron variant in late November 2021, evidence shows that a third shot of vaccine is necessary to restore and sustain protection against severe illness or death [8]. Receiving 3 doses of an mRNA COVID-19 vaccine was associated with a 90% decrease in the risk of invasive mechanical ventilation or mortality. While people who become infected with SARS-CoV-2 following initial immunization often suffer lesser sickness, the protection level against severe illness tends to decline as time passes, given a lack of a booster shot [9].

The effectiveness of booster doses of COVID-19 vaccination against symptomatic illness begins to wane as early as 5 to 9 weeks following the last shot, according to real-world research [10–12]. As the interval between booster doses increases, the protection against Omicron tends to diminish over time [11, 13–15]. Even with the new bivalent booster, vaccine effectiveness after 2 monovalent doses was quite low: 43% among persons aged 18–49 years, 28% among persons aged 50–64 years, and 22% among persons aged 65 years and onward [16]. However, the World Health Organization (WHO) still suggests that protection against serious illness or death is maintained for several months after the third vaccination [8].

The cost-effectiveness of a booster dose has been investigated, although the volume of research on this issue is not huge relative to epidemiological vaccine efficacy studies. Evidence in the United States shows that a third dose booster would incur an additional vaccine cost of US$3.4 million compared to two doses of BNT162b2 without a booster, but would save US$6.7 million in direct medical costs and gain 3.7 quality-adjusted life-years in 180 days. Notably, the cost-effectiveness of the booster method is particularly sensitive to the incidence of COVID-19 in a given period and the effectiveness of vaccination over time [17]. Modelling studies in other countries, such as China and India, demonstrated that all various types of booster vaccines (inactivated vaccines, adenovirus-vectored vaccines, and mRNA vaccines, as well as boosters with fractionated doses) were found to be cost-effective, especially under mass vaccination efforts [18, 19].

Thailand, like other countries, suffered from COVID-19 during 2020–2022, and experienced a surge of cases caused by the widespread of Delta and Omicron variants. By the end of 2022, the number of cumulative cases in Thailand was 4,717,032 with the cumulative death toll at 33,669 [20]. The Thai Government launched a nationwide campaign to encourage people to obtain COVID-19 booster shots to combat the pandemic. The main target population of the booster shot covered the elderly and immunocompromised people, though the general population was also encouraged to receive the booster dose. The Ministry of Public Health (MOPH) provided COVID-19 vaccines free of charge for all Thai residents. Most vaccines in the pool were mRNA (BNT162b2) and viral vector (ChA-dOx1-nCoV) vaccines. Domestic evidence suggested that the third dose of both mRNA and viral vector vaccines offered huge cost-effective benefit in preventing severe illness and fatality to the Thai population [21]. In 2023, the pandemic situation seemed to subside. The Thai Government introduced the concept of “living with COVID” by indicating COVID-19 as an “endemic” disease. Many social measures were lifted, and the booster dose policy still continues to the present but with a lesser degree of campaign intensity. In this regard, the latest Department of Disease Control (DDC) guidelines launched in April 2023 [22] suggests that people should receive an “annual” administration of the COVID-19 vaccine instead of the frequent four monthly booster-shot administration encouraged during the surge of the pandemic in late 2021 (Delta) and early 2022 (Omicron).

A key policy question is whether the current booster dose policy should continue, and whether there are viable policy alternatives with different vaccine administration intervals? If so, which option is the most cost-effective? Therefore, the objective of this study was to assess the cost-effectiveness of the current annual COVID-19 vaccine booster dose policy compared with alternative booster dose policies such as twice-a-year or at a slower biennial pace.

## Methods

### Study design and analysis framework

We combined the concepts of the compartmental susceptible-exposed-infectious-recovered (SEIR) model and the system dynamic (SD) model. Stochastic modelling that accounts for parameter uncertainties was applied [23]. Input for the model was mainly acquired from routine monitoring of the DDC and MOPH.

We focused on Thai adults over 18 years as they are the main target for the Government’s COVID-19 vaccination policy, though children are also allowed to receive the vaccinations with parental permission.

We divided the population into two groups: the young (18–59 years) and older people (at least 60 years). The underlying reason for age-group division is that older people are the priority target for the booster vaccination policy. However, clinical severity and vaccine benefit admittedly vary with respect to age [24, 25]. Then, we classified each group into five strands based on the number of vaccines received: two doses; three doses; four doses; five doses; and six doses or more. The reason for focusing on two-doses upward is because at the time of the study all Thai adults had already received at least two shots of the COVID-19 vaccine. We further applied the SEIR idea to capture the varying degree of epidemic force among the members in each vaccination strand. In each strand, the members were disassembled into five catalogues: the susceptible (infection free); the exposed (infected but not yet infectious); non-isolated infectious (readily in contact with others); isolated infectious referring to either self-isolation at home or hospitalisation (no longer in contact with others); and the recovered (no longer infectious).

**Fig 1:**
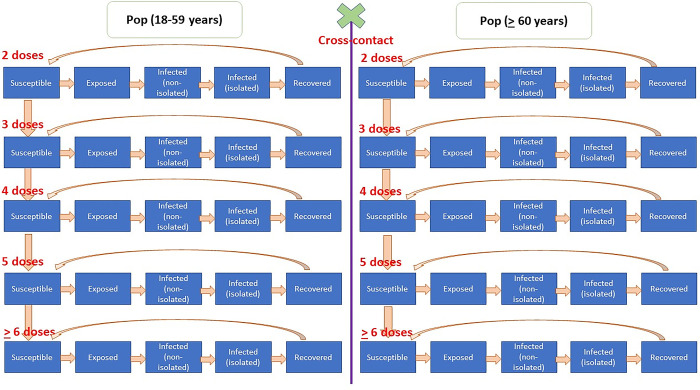
Model framework.

Fig 1 depicts the dynamics of the epidemic via the notion of stocks and flows. The speed of transferring from one stock to another was governed by a differential equation. The prevalent number of people in each stock at a particular point of time was measured by integrating function over the flow. The movement of being susceptible to being exposed was mainly influenced by basic reproductive number (R0). The transition speed from being exposed to being in the non-isolated infectious group was influenced by the SARS-CoV-2 incubation period. The time lag from starting isolation to being isolated was presumed to be four days on average. The recovery time was assumed to vary by clinical severity. The susceptible population moved either to the exposed group or remained in susceptible group when gaining an additional vaccine shot. The rate of crossing from one vaccination strand to another was governed by how frequently a person was supposed to be immunized, which in turn reflects the average vaccination rate in the population.

### Model parameters and assumptions

We used Stella 3.2.1 (registration number: 251-401-786-859) to execute the model. Graphical illustration was performed by Rstudio 4.2.2. The following assumptions were set as grounds for model calculation.

First, the population in each strand was homogenously mixed, implying that all susceptible persons were subject to contracting the disease with equal chance.

Second, we presumed the start date of the model was in early 2023 and the model was run over a four-year course (1460 days).

Third, practically the exact volume of the pool of active infectious population at the start of an outbreak could not be determined. Thus, we presumed that the starting infectious pool size was approximately 14 times the median number of the daily new cases in the first quarter of 2023. The logic behind this is that the volume of the starting infectious pool is likely to equate to a two-week cumulative sum of the daily incident cases. This assumption was also in line with prior experience from the outbreak investigation team of the DDC, in that the notification of the index case for a given local COVID-19 outbreak took place after three to four generations of infective pairs.

Fourth, all symptomatic cases followed isolation (after the time lag), whereas asymptomatic cases did not pass through the isolation process, making them infectious until recovery.

Fifth, we assumed that the influence of the vaccines took place at two points: reducing R0 (according to vaccine effectiveness [VE] against any infection); and altering the clinical severity profile (in line with the VE against severe infection and death, causing a greater proportion of asymptomatic or mildly symptomatic cases and a smaller proportion of severe cases given the increasing number of vaccines).

Sixth, we postulated that the VE waned over 120 days [11, 26, 27]. Moreover, to capture natural immunity after infection, the recovered population would not be infectious for another 120 days. Then, they would return to being susceptible after day 120 (since entering the recovered stock).

Seventh, clinical severity determined treatment modality. Asymptomatic patients needed no treatment (in line with the fourth assumption that they would never be isolated), implying that no treatment cost was incurred. Mild symptomatic patients required outpatient care. Moderate and severe symptomatic patients required hospitalisation. For further specificity, we divided severe cases into those needing intubation and those without.

Eight, we assumed a 20% cross-contact between the young and older people. That is, 80% of the contacts were made within the same age group and 20% of contacts were made across groups.

Ninth, at the time of writing, the number of people receiving more than six vaccination doses was infinitesimal and the effectiveness of the vaccine did not vary much between six doses and afterward. Hence, we proposed a maximum of six doses of vaccination in the model.

Tenth, we assumed all serious adverse events after immunization (AEFI) needed hospital admission with a treatment cost equalling for the cost for treating an inpatient. Other non-serious adverse events underwent spontaneous recovery [28, 29]. Hence, we ignored the treatment cost for non-serious AEFI.

Eleventh, COVID-19 vaccination and treatment are part of the benefit package for all public insurance schemes in Thailand. Thailand has achieved Universal Health Coverage (UHC) for decades. This means, so far, all COVID-19 related costs (in principle) are publicly financed. Thus, the calculation was grounded on public providers’ perspectives (supposing that all cases utilised public services).

Twelfth, we accounted for an annual 3% discount rate for any costs (that is, vaccine administration cost, vaccine vial cost, and treatment cost) and health outcomes involved.

Last, we also accounted for the inflow of infectious people (possibly from tourists or overseas Thais) by assuming 180 asymptomatic cases joining the non-isolated infectious stock each day. This figure was derived from 71,973 tourists entering the country every day [30] and possibly, among these people, 0.25% were asymptomatic cases [31, 32].

We postulated that all model inputs excepting R0 were constant over time. Also, we performed sensitivity analysis by exploring scenarios if R0 was altered as detailed later in the “Interested outcomes and comparative policy options” sub-section.

We calibrated the model to identify optimal R0 by identifying R0 that produced small least square error (while an error identified as difference between the actual number of daily reported cases and the predicted number) during the first two weeks of the model run. We also checked with the DDC monitoring report as to whether the identified R0 matched with the epidemic situation in Thailand at that time.

To account for uncertainty of the model outcomes, we ran the model for over 4,000 simulations applying different values of incubation period, infectious period duration, and isolation time lag.

Simplified key model formulas are depicted in Table 1. Essential parameters of the model are listed in Table 2. The percentage distribution of clinical severity sorted by age groups and varying vaccine effectiveness against hospitalisation [33] and severe illness according to number of doses acquired is detailed in S1 Table.

**Table 1:** Key model formula.

Flow of stock	Formula	Note
Susceptible to exposed	-β*(1-VE)*S*I_ni_/P	β = basic reproduction number/infectious period, VE = effectiveness of vaccine against any infection, S = susceptible population, I_ni_ = non-isolated infectious population, P = total population
Susceptible to non-isolated infectious	-αE	α = 1/incubation period, E = exposed population
Non-isolated isolated infectious	- δI_ni_	δ = 1/time lag from non-isolation to isolation, I_ni_ = non-isolated infectious population
Isolated infectious to recovered	-ζI_i_	ζ = 1/recovery time (depending on clinical severity); I_i_ = isolated infectious population
Susceptible in vaccination strand i to vaccination strand i+1	-νS	ν = 1/vaccination period; S = Susceptible population in lower vaccination strand

**Table 2:** Essential model parameters.

Parameters	Approximated value	Remark or reference	Unit
Basic reproduction number	1.8	Model calibration	Dimensionless
Young population number	40.3*10^^6^	Bureau of Registration Administration, Department of Provincial Administration	Persons
Older population number	12.8*10^^6^	Bureau of Registration Administration, Department of Provincial Administration	Persons
Initial infectious population	10.1*10^^3^	Model assumption	Persons
Intergroup cross-contact percent	20%	Model assumption	Dimensionless
Mean isolation time lag	4.0	Model assumption (assume normal distribution with variance of 0.06)	Days
Mean infectious period	5.1	Wu et al. (assume Gamma distribution with variance of 0.25) [34]	Days
Mean incubation period	2.6	Ogata and Tanaka (assume Gamma distribution with variance of 0.01) [35]	Days
Starting percentage of the young receiving up to second dose^§^	42.1%	Division of Communicable Diseases, Department of Disease Control	Dimensionless
Starting percentage of the young receiving up to third dose^§^	44.6%	Division of Communicable Diseases, Department of Disease Control	Dimensionless
Starting percentage of the young receiving up to fourth dose^§^	11.4%	Division of Communicable Diseases, Department of Disease Control	Dimensionless
Starting percentage of the young receiving up to fifth dose^§^	1.8%	Division of Communicable Diseases, Department of Disease Control	Dimensionless
Starting percentage of the young receiving at least sixth dose^§^	0.1%	Division of Communicable Diseases, Department of Disease Control	Dimensionless
Starting percentage of older people receiving up to second dose^§^	47.4%	Division of Communicable Diseases, Department of Disease Control	Dimensionless
Starting percentage of older people receiving up to third dose^§^	39.1%	Division of Communicable Diseases, Department of Disease Control	Dimensionless
Starting percentage of older people receiving up to fourth dose^§^	11.4%	Division of Communicable Diseases, Department of Disease Control	Dimensionless
Starting percentage of older people receiving up to fifth dose^§^	2.0%	Division of Communicable Diseases, Department of Disease Control	Dimensionless
Starting percentage of older people receiving at least sixth dose^§^	0.1%	Division of Communicable Diseases, Department of Disease Control	Dimensionless
Vaccine effectiveness among the second-dose young vaccinees^±^	47.0%	Modified from Intawong et al. [36]	Dimensionless
Vaccine effectiveness among the third-dose young vaccinees^±^	55.0%	Modified from Intawong et al. [36]	Dimensionless
Vaccine effectiveness among the fourth-dose young vaccinees^±^	62.0%	Modified from Intawong et al. [36]	Dimensionless
Vaccine effectiveness among the fifth-dose young vaccinees^±^	71.0%	Modified from Intawong et al. [36]	Dimensionless
Vaccine effectiveness among the sixth-dose young vaccinees^±^	81.0%	Modified from Intawong et al. [36]	Dimensionless
Vaccine effectiveness among the second-dose older vaccinees^±^	59.0%	Modified from Intawong et al. [36]	Dimensionless
Vaccine effectiveness among the third-dose older vaccinees^±^	58.0%	Modified from Intawong et al. [36]	Dimensionless
Vaccine effectiveness among the fourth-dose older vaccinees^±^	66.0%	Modified from Intawong et al. [36]	Dimensionless
Vaccine effectiveness among the fifth-dose older vaccinees^±^	75.0%	Modified from Intawong et al. [36]	Dimensionless
Vaccine effectiveness among the sixth-dose older vaccinees^±^	85.0%	Modified from Intawong et al. [36]	Dimensionless
Recovery time among asymptomatic cases	5.0	Treatment Guideline of the Department of Medical Services [37]	Days
Recovery time among mild symptomatic cases	5.0	Treatment Guideline of the Department of Medical Services [37]	Days
Recovery time among moderate symptomatic cases	7.0	Assume 1.4–1.5 times the recovery period for the mild	Days
Recovery time among severe symptomatic cases [either with or without intubation]	14.0	Assume 2–3 times the recovery period for the mild	Days
Vaccination administrative cost	234.0	Sirison et al. [21]	Baht
Vaccine vial cost—third dose^*#*^	398.0	Sirison et al. [21]	Baht
Vaccine vial cost—fourth dose^*#*^	488.0	Sirison et al. [21]	Baht
Vaccine vial cost—fifth dose^*#*^	488.0	Sirison et al. [21]	Baht
Vaccine vial cost—sixth dose^*#*^	488.0	Sirison et al. [21]	Baht
Treatment cost for an asymptomatic case	0	Model assumption	Baht
Treatment cost for a mild case	2,550.0	National Health Security Office [38]	Baht
Treatment cost for a moderate case	38,802.0	Wang et al. [38]	Baht
Treatment cost for a non-intubated severe case	95,529.0	Wang et al. [38]	Baht
Treatment cost for an intubated severe case	104,306.0	Wang et al. [38]	Baht
Proportion of persons with adverse events following immunization	0.0083%	Choppradit et al. [39]	Dimensionless

^§^As of first quarter of 2023; ^±^Against any infection; ^#^Weighted average of vial cost between viral vector and mRNA vaccines according to vaccine coverage in the Thai population

### Interested outcomes and comparative policy options

We constructed four policy scenarios which captured the direction of current COVID-19 vaccination policies in Thailand: scenario 1 –a hypothetical scenario where booster immunization is no longer implemented; scenario 2 –a twice-a-year vaccination policy; scenario 3 –an annual vaccination policy; and scenario 4 –a biennial (every-two-years) vaccination policy. In each scenario, the number of doses administered varies depending on the frequency of administration, which can be either 2 doses per year, 1 dose per year, or 1 dose every 2 years for a total of 1460 days (4 years). We assumed 100% coverage of the target population in every booster scenario, and the target population also varies in starting coverage as shown in Table 2.

In each policy scenario, the following outcomes were measured: cumulative total cases (regardless of severity); cumulative admissions (moderate and severe cases combined); cumulative severe cases; and cumulative grand cost (vaccination cost and treatment cost combined).

We later calculated the incremental cost effectiveness ratio (ICER) of SC2-4 with reference to SC1 as per the following formula: ICER = (grand cost_SC[__2__–__4__]_−grand cost_SC1_)/(outcome_SC1_ –outcome_SC[__2__–__4__]_). The final outputs are median and with a 95% confidence limit of ICER for a case averted, ICER for an admission averted, and ICER for a severe case averted; the smaller the value, the more cost effective.

To reflect potential situations given the resurgence of the pandemic, sensitivity analysis was performed by adjusting R0 from 1.8 to 3.0 from day 366 and onward in all scenarios. In addition, we conducted another sensitivity analysis by reducing the vaccination cost (administrative and vial costs) by half after the first year to capture the current policy direction of the Thai Government, which plans to enhance domestic vaccine production to substitute vaccine importing.

### Ethical consideration

As we solely depended on secondary data from international literature and the internal databases of official agencies in Thailand, no human participation was involved. This study is part of the routine monitoring of the policies as per the DDC mandate. In this respect, ethics approval was not required. However, we strictly followed the principles of ethical standards as per the Declaration of Helsinki. No individual information was unveiled.

## Result

**Fig 2:**
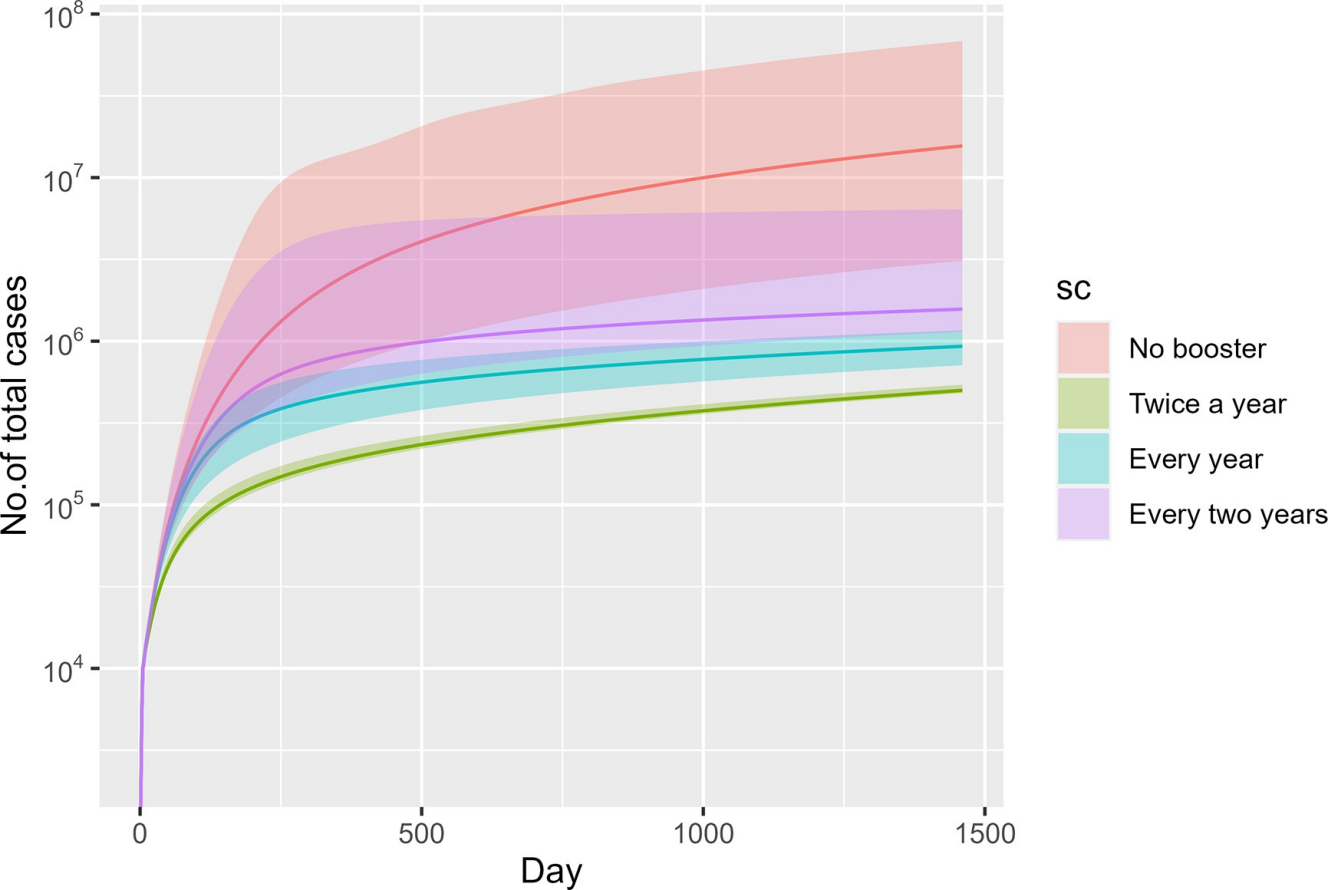
Total cases across time in different scenarios (median and 95% CI).

Fig 2, from a macro perspective, shows the total number of cases in different scenarios. The median number of total cases of scenario 1 (no booster vaccination) was higher than all other booster scenarios. The volume of total cases in a no booster scenario would reach 16,220,615 (95% CI 6,726,550–29,684,334) by day 1460. In a vaccine booster scenario, the number of total cases would vary depending on vaccination intervals. The number of total cases would be lowest in scenario 2 (twice-a-year booster), followed by scenario 3 (annual booster) and scenario 4 (biennial booster). By day 1460, the total cases in a twice-a-year booster scenario would amount to 597,901 (95% CI 526,230–694,458), while for annual boosters and biennial boosters, the number would reach 971,043 (95% CI 853,840–1,152,030) and 1,444,150 (95% CI 982,825–2,448,443), respectively. We presented the number of total cases in a natural log scale in order to account for a vast range of numbers between different scenarios.

**Fig 3:**
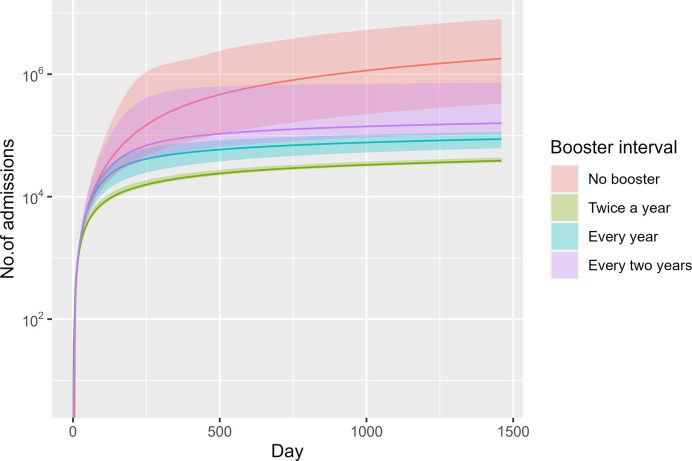
Total admissions across time in different scenarios (median and 95% CI).

Fig 3 displays the admissions toll in different scenarios. The pattern of admissions number was similar to Fig 2. The median number of admissions in no booster scenario was larger than the booster scenarios. Total admissions in no booster scenario would reach 1,870,971 (95% CI 757,160–3,450,740) in the next four years. By day 1460, the cumulative admissions toll in the twice-a-year booster would be about 48,628 (95% CI 40,246 – 59,922). The biennial booster policy would result in 143,090 (95% CI 89,052–260,738) admissions by day 1460, whereas the annual booster policy results lay approximately between the twice-a-year and the biennial booster policies.

**Fig 4:**
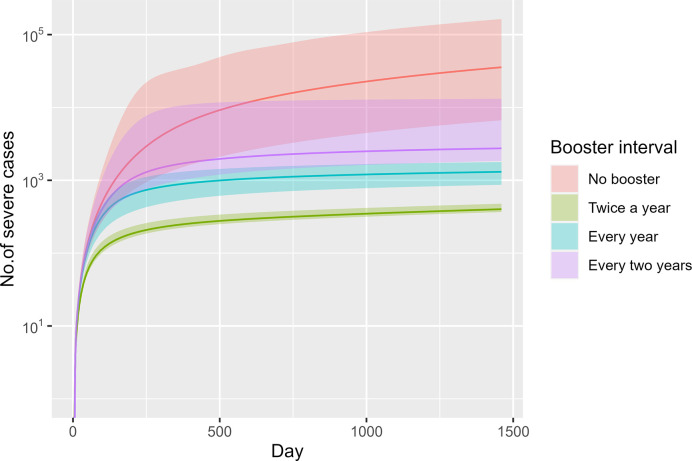
Total severe cases across time in different scenarios (median and 95% CI).

The volume of severe cases in a no booster scenario would exceed 30,000 by about day 1,200. In the twice-a-year booster scenario, the projected number of severe cases is 559 (95% CI 426–740) by the end of the time horizon. That is approximately one third of the total in the annual booster scenario, which comprises 1,368 (95% CI 1,130–1,739), and approximately one fifth of the total in the biennial booster scenario, amounting to 2,468 cases (95% CI 1,493–4,605) (Fig 4).

Regarding healthcare and vaccination costs, no booster scenario exhibited the lowest cost during the first 500 days by approximation, then it skyrocketed and outstripped the biennial scenario. By the end of the analysis horizon, the twice-a-year booster scenario would incur the highest cost with a median of approximately 121.7 billion baht (95% CI 121.2–122.4 billion).

**Fig 5:**
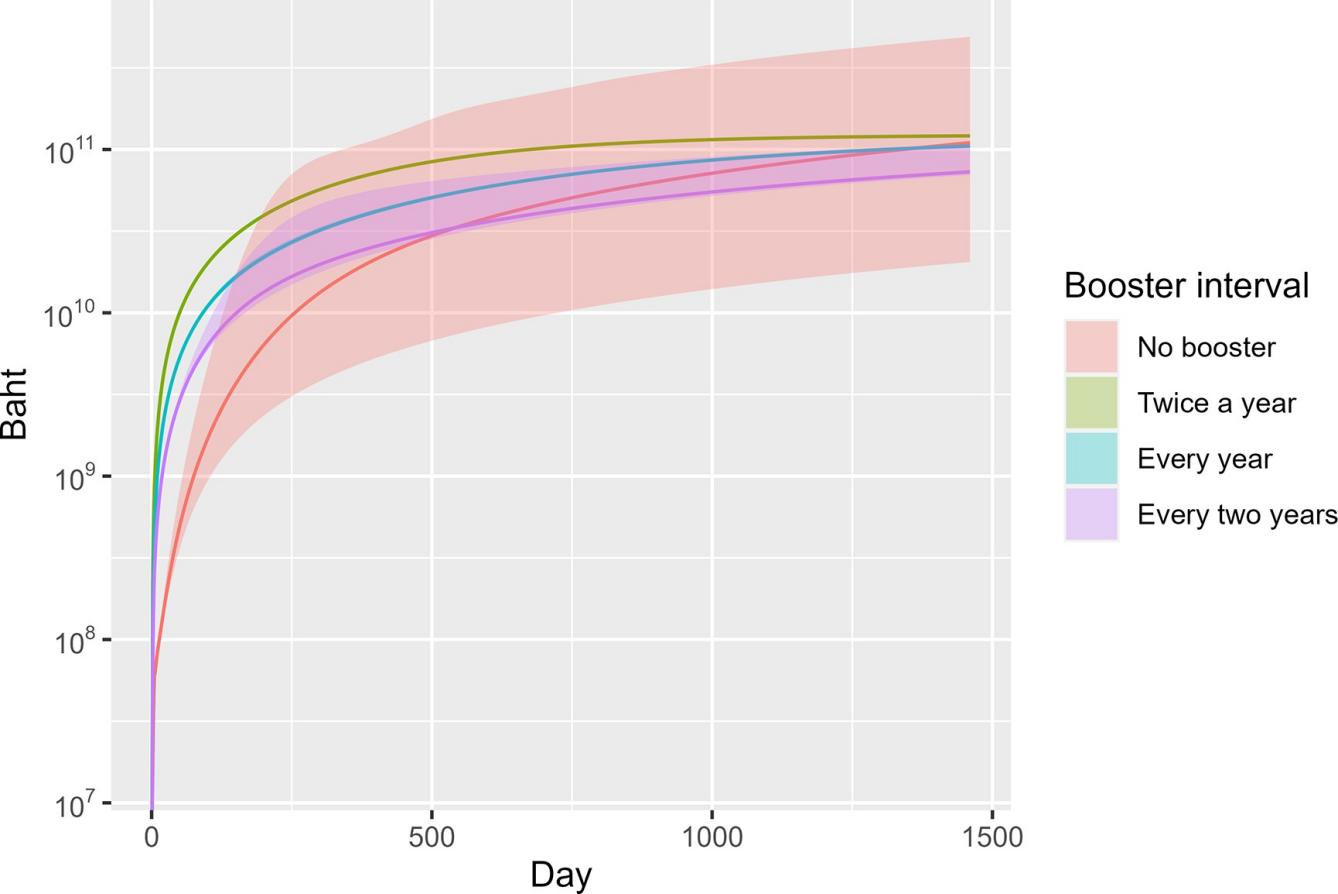
Grand cost across time in different scenarios (median and 95% CI).

The no booster scenario came second at 114.3 billion baht (95% CI 46.3–210.9 billion), followed by the annual booster scenario at 105.2 billion baht (95% CI 104.3–106.5 billion). By the last day of the calculation period, the biennial booster scenario demonstrated the lowest cost at 72.0 billion baht (95% CI 68.6–79.4 billion) (Fig 5).

By considering the no booster scenario as a reference, the biennial booster scenario was the most cost-effective policy option with a negative ICER regardless of the concerned outcomes implying that biennial booster scenario became cost-saving. The annual booster scenario presented as a cost-effective option, despite not cost-saving. For the twice-a-year booster, it incurred 2,963 baht for each case averted, 25,595 baht for an admission averted, and 1,268,027 baht for a severe case averted (Table 3).

**Table 3:** Incremental cost effectiveness ratio analysis (main assumption).

ICER by day 1,460	Comparison scenario (ref = no booster)	95% CI lower limit	Median	95% CI upper limit
Baht per a case averted	Twice-a-year booster	-3,242	2,963	13,296
	Annual booster	-3,895	1,588	11,307
	Biennial booster	-5,225	-1,584	7,973
Baht per an admission averted	Twice-a-year booster	-27,717	25,595	114,968
	Annual booster	-33,284	13,747	97,670
	Biennial booster	-44,609	-13,506	68,691
Baht per a severe case averted	Twice-a-year booster	-1,381,928	1,268,027	5,632,669
	Annual booster	-1,658,481	676,533	4,761,979
	Biennial booster	-2,226,745	-671,519	3,292,342

In a situation where the epidemic was more intense, all booster programs became cost-saving. With reference to a no booster scenario, all other booster policies produced fairly similar results irrespective of the outcomes.

For cumulative case toll and cumulative admissions, biennial booster scenario demonstrated the most negative ICER by saving 7,010 baht per case averted (95% CI [–7,109]–[–6,878]) and 56,616 baht per admission averted (95% CI [–60,434]–[–58,506]), outperforming the annual booster scenario with an extremely close margin.

Focusing on severe cases, the annual booster scenario saved approximately 2,475,874 baht per severe case averted (95% CI [–2,535,903]–[–2,419,236]). The biennial booster scenario also remained the most cost-saving option, albeit with an extremely small margin, in terms of case aversion and admission aversion, relative to the annual and twice-a-year booster scenarios (Table 4).

**Table 4:** Incremental cost effectiveness ratio analysis (R0 = 3.0).

ICER by day 1,460	Comparison scenario (ref = no booster)	95% CI lower limit	Median	95% CI upper limit
Baht per a case averted	Twice-a-year booster	-7,109	-7,010	-6,878
	Annual booster	-7,066	-6,951	-6,788
	Biennial booster	-7,236	-7,060	-6,595
Baht per an admission averted	Twice-a-year booster	-60,434	-59,616	-58,506
	Annual booster	-60,047	-59,077	-57,705
	Biennial booster	-61,470	-59,944	-55,897
Baht per a severe case averted	Twice-a-year booster	-2,626,007	-2,563,464	-2,506,759
	Annual booster	-2,535,903	-2,475,874	-2,419,236
	Biennial booster	-2,581,518	-2,390,267	-2,037,284

In an assumption where vaccination costs dropped by 50% after the first year, all booster scenarios became more cost-effective or cost-saving compared with the initial assumption. The biennial booster scenario stood out as the most cost-saving by saving 3,221 baht per a case averted, 27,538 baht per an admission averted, and 1,370,653 baht per a severe case averted. The annual booster scenario ranked as the second option by saving approximately a quarter of the biennial booster scenario. Following this was the twice-a-year booster scenario (Table 5).

**Table 5:** Incremental cost effectiveness ratio analysis (50% reduction of vaccination cost).

ICER by day 1,460	Comparison scenario (ref = no booster)	95% CI lower limit	Median	95% CI upper limit
**Baht per a case averted**	Twice-a-year booster	-4,230	556	8,522
	Annual booster	-4,864	-829	6,322
	Biennial booster	-5,867	-3,221	3,749
**Baht per an admission averted**	Twice-a-year booster	-36,161	4,890	73,686
	Annual booster	-41,562	-7,024	54,607
	Biennial booster	-50,088	-27,538	32,296
**Baht per a severe case averted**	Twice-a-year booster	-1,802,916	238,813	3,610,094
	Annual booster	-2,070,960	-353,944	2,662,377
	Biennial booster	-2,500,258	-1,370,653	1,547,955

## Discussion

Overall, we found that the twice-a-year booster policy with the shortest interval (six months) outperformed other booster policies in averting the case burden, but at the same time, incurred the highest amount of cost involved. Interestingly, the hypothetical no booster scenario caused the lowest cost approximately in the first 500 days, but after that, the biennial scenario bore the lowest cost. This points to the fact that the COVID-19 vaccination is still worth investment.

Considering the ICER analysis, the biennial policy was the most cost effective, followed by the annual booster policy. Given a high epidemic situation and in the situation where vaccination cost declined, all booster policies became cost-saving (as confirmed by negative ICER) and produced relatively similar ICER. The annual booster policy produced almost the same results as the biennial booster policy. In a scenario where vaccination costs dropped, all policies became more cost-effective or cost-saving compared with before the decline in the vaccination cost. The biennial booster scenario was the most cost-saving approach in all outcomes.

Our findings concur with many studies abroad [17–19]. For instance, Fu et al suggested that COVID-19 vaccination and booster vaccination were cost-effective or cost-saving independent of the vaccine types. Moreover, they pointed that vaccine price, vaccine supply, and vaccination pace are influential factors that determine the level of cost-effectiveness [40]. In the U.S., every dollar spent on the BNT162b2 booster vaccination after the initial two doses reduces COVID-19 hospitalizations by nearly US$ 2. In addition, they suggested that the booster vaccine would be cost-effective at an incidence threshold of at least 8.1/100,000 persons per day [17]. As well as the fractional ChAdOx1 nCoV-19 booster in India, it has been demonstrated that under the effective reproduction number of 1.2 and of 5 transmission scenarios, the estimated health-related net monetary benefit over 200 days is US$6 billion and US$2 billion, respectively [19].

Thailand’s recommendation for a yearly booster shot is consistent with the United States Food and Drug Administration (FDA) and the United States Centers for Disease Control and Prevention (CDC) discussions [41–44]. Even though the CDC’s current recommendation suggests an additional booster only for those who are elderly or immunocompromised, it intends to simplify the booster schedule for all people [45]. The Vaccines and Related Biological Products Advisory Committee (VRBPAC) of the FDA also recommended the annual immunization schedule at its January 2023 meeting [46, 47]. In Canada [48] and the UK [49], certain populations at high risk of severe COVID-19, including older people, people in long-term care facilities, and immunocompromised individuals, began receiving an additional booster dose.

Our study demonstrated that the biennial policy was the most cost-effective, especially when the disease was in a state of low epidemic. Nevertheless, the annual booster policy was more practical in terms of implementation feasibility and communication simplicity. The same idea was applied with the influenza vaccine where the annual booster dose is universally accepted. Moreover, it is possible that healthcare providers can administer the influenza vaccine and COVID-19 vaccine at the same time. The FDA also proposes annual COVID boosters, similar to annual flu shots [41, 43]. FDA experts also suggest simplifying annual booster immunization in order to facilitate vaccine deployment and minimize vaccine administration errors, all of which can potentially lead to improved vaccine coverage in the population [46]. In addition, the sensitivity analysis demonstrated that a shorter interval of booster dose would be more cost-effective at higher epidemic levels. Similarly, in the event of measles outbreak, the mass (booster) vaccination, in addition to the regular schedule, is recommended [50]. Therefore, considering the current epidemic circumstances, either the annual booster policy or biennial policy is an optimal immunization choice for the whole population; however, the annual booster policy is more advantageous in terms of management.

This study had both strengths and limitations. Regarding strengths, we included a large number of parameters, most of which were based on real-world data. We also performed a sensitivity analysis to assess whether the cost-effectiveness level would change the policy choice, given different epidemic stages and different vaccine investment costs.

However, some limitations remained. Firstly, many parameters were based on an underlying assumption that they were not time dependent. For example, the VE might diminish when SARS-CoV-2 undergoes genetic mutation.

Secondly, most variables solely reflected disease dynamics, but in reality, they depend on healthcare system functioning. For instance, the percentage distribution of clinical severity is not a matter of the pathological profile of the disease alone as it relies on the performance of clinical care. In a high epidemic stage, the quality of care may be compromised due to an overwhelming number of cases exceeding care capacity [51, 52]. Besides, the reporting system did matter. In late 2022, the ‘living with COVID’ policy resulted in a significant change in the reporting system of COVID-19 cases by the Thai health facilities. This also led to a huge fluctuation in the number of cases reported to the DDC in late 2022. We hence decided not to include data in 2022 for model calibration despite a trade-off of having fewer data points. However, we deemed that the model usefulness is still intact since the primary aim of the model was more of presenting input for policy decision under difference vaccination scenarios rather than offering the most accurate forecast to aide resource planning for pandemic response.

Thirdly, we were unable to measure the impact of COVID-19 booster shots in terms of years gained or quality-adjusted life years (QALY) due to the limited availability of data on QALY gained from vaccine boosters in Thailand. Nonetheless, the primary focus of the DDC policy is on the burden of cases in seasonal epidemics and the severity of their outcomes [22], rather than QALY. Though long-term COVID-19 is of concern, the policy focus so far has been centered on the observed short-term impact of COVID-19 infection.

Fourthly, a considerable portion of the variables employed in our study comprises internal data that do not have clear information on the data distribution nature. Consequently, comprehensive probability sensitivity analysis that accounted for the distribution nature of all variables was precluded. This issue also points to a dire need for further research to explore the nature of COVID-19 related data. Repeating this kind of study when relevant data are updated is recommended.

Finally, this study uses the Thai healthcare system and epidemic profile as a case study. The application of the results in different contexts should be made with caution as decisions on COVID-19 booster policy do not rely on the matter of cost-effectiveness alone. Many other factors that were not included in this study such as budget impact, vaccine prioritization, and willingness to pay always play a pivotal role in policy decision-making. These issues also warrant further studies.

## Conclusion

This study reaffirms the importance and the merit of an evidence-based COVID-19 vaccine booster dose policy. In a low epidemic stage, the annual and the biennial policies would be a cost-effective investment in preventing overall infection in the population, as well as hospitalization and severe infection. In a situation where the epidemic was intense or when the cost of vaccines reduced, a shortening of vaccination interval—either every six months or once a year—would be worthy of investment close to the biennial booster policy. The implementation of any booster policies should consider not only the issue of cost effectiveness but also other managerial factors, such as policy feasibility and simplicity in public communication. Further studies on budget impact and strategies for vaccine prioritization are recommended.

## Supporting information

S1 Table Percentage distribution of clinical severity sorted by age groups and number of vaccines shots. (DOCX)
