# Supplementary material for: Cost effectiveness analysis comparing varying booster intervals of vaccination policies to address COVID-19 situation in Thailand, 2023
Source: PLoS One. 2024 Sep 17;19(9):e0310427. doi: 10.1371/journal.pone.0310427 (PMC11407652; doi:10.1371/journal.pone.0310427)
Supplement: S1 Table — (DOCX) [file pone.0310427.s001.docx]

**S1 Table.** **Percentage distribution of clinical severity sorted by age groups and number of vaccines shots**

| Number of doses | Age groups | Clinical severity | Percentage |
| --- | --- | --- | --- |
| 2 doses | 18-59 years | Asymptomatic | 38.93% |
|  |  | Mild | 49.40% |
|  |  | Moderate | 11.51% |
|  |  | Severe with no intubation | 0.08% |
|  |  | Severe with intubation | 0.07% |
|  | >60 years | Asymptomatic | 38.93% |
|  |  | Mild | 48.37% |
|  |  | Moderate | 11.27% |
|  |  | Severe with no intubation | 0.84% |
|  |  | Severe with intubation | 0.60% |
| 3 doses | 18-59 years | Asymptomatic | 38.93% |
|  |  | Mild | 49.47% |
|  |  | Moderate | 11.53% |
|  |  | Severe with no intubation | 0.04% |
|  |  | Severe with intubation | 0.03% |
|  | >60 years | Asymptomatic | 38.93% |
|  |  | Mild | 48.98% |
|  |  | Moderate | 11.41% |
|  |  | Severe with no intubation | 0.42% |
|  |  | Severe with intubation | 0.26% |
| 4 doses | 18-59 years | Asymptomatic | 38.93% |
|  |  | Mild | 49.49% |
|  |  | Moderate | 11.53% |
|  |  | Severe with no intubation | 0.03% |
|  |  | Severe with intubation | 0.01% |
|  | >60 years | Asymptomatic | 38.93% |
|  |  | Mild | 49.04% |
|  |  | Moderate | 11.43% |
|  |  | Severe with no intubation | 0.45% |
|  |  | Severe with intubation | 0.15% |
| 5 doses | 18-59 years | Asymptomatic | 38.93% |
|  |  | Mild | 49.49% |
|  |  | Moderate | 11.53% |
|  |  | Severe with no intubation | 0.03% |
|  |  | Severe with intubation | 0.01% |
|  | >60 years | Asymptomatic | 38.93% |
|  |  | Mild | 49.02% |
|  |  | Moderate | 11.42% |
|  |  | Severe with no intubation | 0.53% |
|  |  | Severe with intubation | 0.09% |
| 6 doses | 18-59 years | Asymptomatic | 38.93% |
|  |  | Mild | 49.47% |
|  |  | Moderate | 11.53% |
|  |  | Severe with no intubation | 0.04% |
|  |  | Severe with intubation | 0.02% |
|  | >60 years | Asymptomatic | 38.93% |
|  |  | Mild | 49.12% |
|  |  | Moderate | 11.45% |
|  |  | Severe with no intubation | 0.25% |
|  |  | Severe with intubation | 0.25% |
